# Making the Public Understand

**Published:** 1920-09-04

**Authors:** 


*To the Editor of* THE HOSPITAL.

### Making the Public Understand.

*To the Editor of* THE HOSPITAL.

SIR,—In her letter published in the current number of THE HOSPITAL, "A College Member" commenting on my article "The Way of Attainment" asks: "Who are our leaders in the profession?" This is a difficult question to answer since the attributes which combine to form the personality of a leader may be entirely missing in that of a representative. Yet if we read the names of the members of the Council of the College of Nursing and the General Nursing Council several stand out pre-eminent, not because they are matrons of large training-schools, but because they have inspired confidence in the rank-and-file of the nursing services and have identified themselves with a definite policy of progress.

As honorary press secretary to a local centre I can assure "A College Member" it is extremely difficult to obtain a hearing for the nursing services in the columns of the daily press; space is tight, and editors though courteous are not interested in a subject of which they are ignorant, and in which they have no reason to believe the public are better informed than themselves. We are now paying for our past mistakes, and if we are to take that place which we have earned in the national life of the people we must first educate public opinion by means of propaganda. The crying need of the moment is "A Publicity Department," but the questions immediately arise, Who is to bear the expense and what scheme of co-ordination has been evolved for public consideration? These are pertinent questions and they do not admit of frivolous answers, they are of vital importance to the future of the nursing services.—Yours faithfully,

THE WRITER OF "THE WAY OF ATTAINMENT."

